# Salivary alpha‐synuclein as a potential fluid biomarker in Parkinson’s disease: A systematic review and meta‐analysis

**DOI:** 10.1002/agm2.12192

**Published:** 2022-01-24

**Authors:** Sanjeev Kharel, Rajeev Ojha, Anil Bist, Surya Prakash Joshi, Robin Rauniyar, Jayant Kumar Yadav

**Affiliations:** ^1^ Maharajgunj Medical Campus Tribhuvan University Institute of Medicine Kathmandu Nepal; ^2^ Department of Neurology Tribhuvan University Institute of Medicine Kathmandu Nepal; ^3^ Department of Internal Medicine Tribhuvan University Institute of Medicine Kathmandu Nepal

**Keywords:** biomarker, oligomeric, Parkinson, salivary alpha‐synuclein

## Abstract

**Introduction:**

Lewy bodies are the pathological hallmarks of Parkinson's disease. There is a need for effective biomarker that is cost effective, less invasive, and easily reproducible with good sensitivity and specificity and can be used to diagnose the condition early and track its severity and progression. Alpha‐synuclein (α‐syn), an integral component of the Lewy body, is found in saliva and can be a potential answer to the above concerns.

**Methods:**

PubMed, EMBASE, Google Scholar, and CNKI databases, along with additional sites, were searched from January 2010 to August 2021. Standard mean difference (Hedges' g) with 95% CI was used to show an association. Statistical analysis was done using STATA software version 16 (StataCorp).

**Results:**

We found a significant reduction in the mean difference of total salivary α‐syn among PD patients compared to healthy controls. However, the mean difference of oligomeric α‐syn and oligo/total salivary α‐syn ratio was significantly increased among PD patients compared to healthy controls.

**Conclusion:**

Our systematic review and meta‐analysis found that salivary α‐syn parameters (total, oligomeric, oligo/total) can be considered a simple, easy‐to‐use, cost‐effective, and reliable diagnostic biomarker for PD and its progression.

## Graphical abstract

Alpha‐synuclein parameters (total, oligomeric, oligo/total) can be considered as a simple, easy to use, cost‐effective, and reliable diagnostic biomarker for PD and its progression.

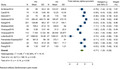

## 1 INTRODUCTION

Parkinson's disease is a common progressive neurodegenerative disease. Degeneration of dopaminergic neurons in the substantia nigra pars compacta and the formation of intracytoplasmic inclusions known as Lewy bodies (LB) are pathological hallmarks of Parkinson's disease.^1^ The diagnosis is clinical, based on the presence of rest tremors, rigidity, bradykinesia, and loss of postural reflexes, which are cardinal signs of Parkinson's disease.^2^ However, a significant portion of nigral neurons is lost prior to the onset of motor symptoms. Consequently, the clinical diagnosis often occurs too late to administer any disease‐modifying therapies that will help salvage the remainder of the dopamine‐producing neurons. Hence, there is a need for effective biomarker that is cost‐effective, less invasive, and easily reproducible with good sensitivity and specificity and can be used to diagnose the condition early and track its severity and progression. However, the search for such a biomarker has been elusive until now.

Alpha‐synuclein(α‐syn) aggregates are found in LB. The accumulation causes neuronal dysfunction and neuroinflammation. It has been shown that microglia surround a‐syn aggregates and when activated can cause increased extracellular superoxide, inflammatory cytokines, and selective loss of dopaminergic neurons.^3^ Alpha‐synuclein is both genetically and pathologically linked to PD. Similarly, neuronal death in PD is resulted by oligomerization of a‐syn. Thus, identification of these early aggregates may help in the early detection of disease and serve as promising biomarkers.^4^ Salivary glands are associated with LB pathology in the early stages of Parkinson's disease.^5^ Alpha‐synuclein is found in nerve fibers innervating salivary glands. Similarly, submandibular gland biopsies among PD patients showed positive staining for α‐syn, providing solid evidence for saliva as a diagnosing PD biomarker.^6^ Furthermore, salivary samples are cost effective and relatively safe with a less invasive collection process and low blood contamination levels.

Previous studies showed varying total salivary α‐syn levels in PD patients compared to controls. Several studies have shown significantly lower total salivary α‐syn levels in PD patients compared to controls.^7^, ^8^, ^9^, ^10^ While other studies showed no significant changes in total, salivary α‐syn levels.^6^, ^11^, ^12^, ^13^, ^14^ Further, differences in subtypes of α‐syn levels, including oligomeric α‐syn levels, have been found between PD and controls and can be used as a biomarker. Thus, this warrants a need for robust analysis to show the exact relationship between salivary α‐syn levels between PD and controls and its utility as an effective biomarker.

## 2 METHODS

This systematic review and meta‐analysis are reported according to the PRISMA (Preferred Reporting Items for Systematic Reviews and Meta‐Analyses) following the PRISMA checklist and flow diagram for manuscript format development.^15^ The first step of this review involved forming the research question. Our question was whether salivary α‐syn in PD patients might differ significantly from healthy controls. Next, we drafted the inclusion and exclusion criteria for study selection.

**Study inclusion and exclusion criteria:**

The inclusion criteria were as follows:

Study type(s): Prospective or retrospective studies published in any language were considered eligible to be included in this review.
Study cases(s): Subjects with PD of any age, gender, or nationality whose saliva was evaluated using any available techniques were eligible.
Study control(s): Subjects of any age, gender, or nationality without PD and other diseases whose saliva was evaluated using any available techniques were eligible.
Objective outcome(s): Studies should at least compare total salivary alpha‐synuclein between cases and controls. Additional outcomes included but not mandatory were (1) oligomeric salivary alpha‐synuclein (2) ratio between oligomeric to total salivary alpha‐synuclein.

‐ Study result (s): Studies providing adequate data for calculations of mean difference of salivary alpha‐synuclein between cases and control and its 95% confidence interval were included.

The exclusion criteria were as follows:

Study with insufficient information.
Animal studies.
Review articles, case reports/series, one‐arm clinical trials, conference abstracts
Studies not reporting our primary outcome.

### 2.1 Search methods

PubMed, EMBASE, Google Scholar, and CNKI databases were searched for any language literature from January 2010 to August 2021. Boolean logic was used for conducting a database search, and Boolean search operators "AND" and "OR" were used to link search terms. Search strategy for PubMed search was as follows: ("Parkinson Disease"[MeSH Terms] OR "Parkinsonian Disorders"[MeSH Terms]) AND (("alpha‐Synuclein"[MeSH Terms] AND ("Saliva"[All Fields] OR "Salivary gland"[All Fields])) OR ("salivary"[All Fields] AND ("alpha‐Synuclein"[MeSH Terms] OR "alpha‐Synuclein"[All Fields] OR ("alpha"[All Fields] AND "synuclein"[All Fields]) OR "alpha‐Synuclein"[All Fields]))). Search strategy for EMBASE was as made with the help of emtree terms. The detailed search strategy is given in the supplementary file, Appendix S1. A search for grey literature was also conducted in the Open grey site. The articles on West Pacific Index Medicus (WPRIM) were also searched and included if relevant. The search was also broadened to include conference proceedings in journals, preprint servers, and thesis repositories. We surfed the reference list of each included study to identify other potential material of interest.

### 2.2 Study selection

Two independent investigators (SK and AB) performed a literature search from databases and additional sources. All the results were exported into the reference manager software, EndNote X7 (Thomson Reuters, New York, NY, USA). The studies were screened by abstract, and titles and duplicates were removed. Two investigators shortlisted articles after applying the inclusion and exclusion criteria. The third author (RO) came into play in case of any disagreements. An overall evaluation for potential overlap of the population was conducted based on authorship, hospital setting, and recruitment period. In cases of overlap, higher‐quality studies or larger sample sizes were included.

### 2.3 Data extraction

Two independent investigators (SK and AB) extracted data in an excel spreadsheet (Microsoft Corp.) using a standardized data extraction form. Results were compiled to complete the following fields: Author, year of publication, study site, study design, number of patients (PD and controls), measurement method, the mean age of patients and controls, the sex ratio of patients and controls, and the disease duration. The mean total salivary alpha‐synuclein among PD and controls was extracted. Data from other outcomes like oligomeric salivary alpha‐synuclein and ratio of oligomeric to total salivary alpha‐synuclein were also extracted among PD patients and healthy controls. A third reviewer (RO) was consulted to resolve inconsistencies when consensus was not reached. If the required data were missing, not reported in the paper, or reported in an unusual form, the corresponding authors of the respective papers were contacted via email for clarification. Supplementary Material associated with the main paper was also explored in such cases.

### 2.4 Quality appraisal

Two investigators (SK and AB) evaluated the quality of included studies in a consensus procedure. The Newcastle‐Ottawa Scale (http://www.ohri.ca/programs/clinical_epidemiology/oxford.asp) was used for the quality assessment of each study and described under three headings: selection,^5^ comparability,^2^ and exposure.^3^ Two authors independently assessed the study while any disagreements were solved through discussion and the third author. Studies with scores of five or higher qualified for inclusion, while scores more than seven were considered high‐quality studies.

### 2.5 Statistical analysis

All statistical analysis was performed using the STATA software version 16 (StataCorp). The pooled mean differences of salivary alpha‐synuclein (total, oligo, oligo to total) between PD patients and healthy controls were evaluated using Standard Mean Difference (Hedges' g) with 95% CI. For those studies reporting median, range, and interquartile range, mean and SD were calculated.^16^ A random‐effects or fixed‐effect model was used to pool the data, and statistical heterogeneity was evaluated using the *I*² statistic. When *I* ^2^ was ≤50%, a fixed‐effect model was used for meta‐analysis. When *I* ^2^ was >50%, DerSimonian and Laird's random‐effects model was used for meta‐analysis. Forest plots with 95% CIs were created to show overall weighted mean estimates with 95% CI. A *p*‐value of <0.05 was considered statistically significant.

### 2.6 Sensitivity analysis and publication bias

Sensitivity analysis was performed by sequentially omitting one study at a time to check the robustness of the analysis. The publication bias was assessed by Begg's test for small‐study effect size and shown in a funnel plot of standard error and effect size. A *P*‐value of <0.05 was considered statistically significant.

## 3 RESULTS

### 3.1 Search results and study characteristics

**FIGURE 1:**
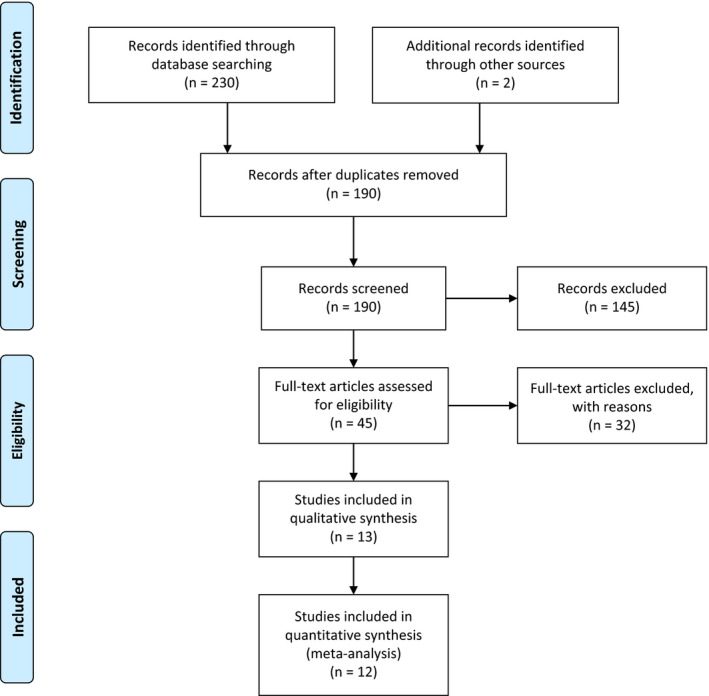
Prisma diagram showing results of our literature search and selection

In total, 230 articles were identified after a thorough database search published from 2010 to 2021. Forty studies were removed as duplicates, and titles and abstracts screened 190. Out of the remaining 45 studies, 32 were excluded. After excluding duplicates and those not meeting inclusion criteria, 13 studies were reviewed for data collection.^6^, ^7^, ^8^, ^9^, ^10^, ^11^, ^12^, ^13^, ^14^, ^17^, ^18^, ^19^, ^20^ Figure 1 shows the results of our literature search and selection. The characteristics of each included study discussed below are summarized in Table 1. Almost all of the included studies were retrospective. Of the 13 studies, 11 studies were in the English language, and 2 were exclusively in the Chinese language. Four studies were conducted in China,^6^, ^13^, ^17^, ^20^ three in the USA,^11^, ^12^, ^14^ two in Italy,^8^, ^9^ and one study each was conducted in Iraq,^7^ Egypt,^10^ Spain,^18^ and Japan.^19^

**TABLE 1:** The detailed characteristics of each included study

Author	Year of publication	Study site	Type of study	Number of patients		Method	Mean age		Sex ratio		Duration of PD	Alpha‐synuclein(total)		Alpha‐synuclein(oligo)		Alpha‐synuclein (Oligo/total)	
				PD	HC		PD	HC	PD (F/M)	HC (F/M)		PD	HC	PD	HC	PD	HC
Al‐Nimer	2014	Iraq	CS	20	20	ELISA	64.4 (10.6), 66	65.4 (8.2), 64	4/16 (25%)	2/18 (11.11%)	6.55 ± −6.83 (4)	65 ± 52.2 pg/ml	314.01 ± 435.9	NA	NA		
Cao	2018	China	CS	74	60	ECL (Electro chemiluminescence)	59.62 (8.57)	58.75 (9.85)	34/40 (85%)	34/26 (130.77%)	5.5 (3–7.5)	11.39 (6.23–28.11)	12.23 (5.47–58.83)	10.39 ± 1.46 (pg/ng)	1.37 ± 0.24	1.70 ± 0.52(pg/ng)	0.67 ± 0.26
Devic	2011	USA	CS	24	25	Luminex	63.5 (11.3)	58 (10.4)	7/17 (41.2%)	14/11 (127.3%)	8.5 (6.4)	70 ± 80 pg/ml	110 ± 130	NA	NA		
Goldman	2018	USA	CS	115	88	ELISA	68.24 (6.40)	65.64 (7.36)	43/72 (59.7%)	29/59 (49.2%)	8.34 (3.09)	285.42 ± 400.13 pg/ml	165.97 ± 272.15	NA	NA		
Kang	2016	China	CS	201	67	Luminex	63.18 (9.67)	61.04 (10.01)	79/122 (64.75%)	26/41 (63.41%)	NA	128.66 ± 98.2 pg/ml	131.31 ± 104.1				
Shaheen	2020	Egypt	CS	25	15	ELISA	60.1 (5.6)	60 ± 6.7	10/15 (66.67%)	5/10 (50%)	3.8 ± 2.7	159.4 ± 61.6 ng/ml	229.9 ± 64	47.8 ± 11.8ng/ml	39.2 ± 9.2	0.35 ± 0.18	0.19 ± 0.08
Stewart	2014	USA	CS	24	198	Luminex	63.5 ± 11.3	54.9	7/17 (41.18%)	61/137 (44.53%)	8.4 (6.4)	0.07 ± 0.08 (NA)	0.37 ± 0.02 pg/miug	NA	NA		
Vivacqua	2016	Italy	CS	60	40	ELISA	66.3 (8.78)	68.3 (7.9)	29/31 (93.55%)	18/22 (81.82%)	6.7 (10.4)	5.08 ± 3.01 pg/ml	31.3 ± 22.4	1.062 ± 0.266 ng/ml	0.498 ± 0.203	0.174 ± 0.044	0.065 ± 0.027.
Vivacqua	2019	Italy	CS	112	90	ELISA	69.01 (11.16)	62.09 (15.08)	53/59 (89.83%)	37/53 (69.81%)	NA	7.104 ± 5.122 pg/ml	28.444 ± 25.877	0.893 ± 1.949 ng/ml	0.217 ± 0.191	0.235 ± 0.793	0.013 ± 0.008
Lufen Su	2018	China	CS	27	27	ELISA	61.52 (9.57)	58.37 (10.63)	12/15 (80%)	12/15 (80%)	NA	1269.02 ± 16.09 ng/ml	1350.51 ± 25.79	NA	NA	NA	NA
Fernandez‐Espejo	2021	Spain	CS	45	30	ELISA	61.4 (18.5)	59.6 (11)	18/27 (66.67%)	18/12 (150%)	9.9 ± 6.8	361.9 ± 89 pg/ml	372.1 ± 91				
Kawabe	2013	Japan	CS	20	20	Restriction Enzyme Analysis	NA	NA	NA	NA	NA	78.9 ± 91.9 pg/ml	158.1 ± 71.7				
Pang	2016	China	CS	38	21	Enzymatic cholorimetry	68.3 (10.6)	60.5 (11.4)	17/21 (80.95%)	12/9 (133.3%)	NA	1.35 ± 9.87 U/L	1.63 ± 1.21				

Abbreviations: CS, Cross‐sectional; ELISA, enzyme‐linked immunosorbent assay; HC, Healthy controls; PD, Parkinson disease.

### 3.2 Demographics, duration of PD, methods of measurement of salivary alpha‐synuclein, outcomes

In 13 studies, the sample of PD patients ranged from 20 to 201. The sex ratio (female/male) of PD patients ranged from 25% to 93.55% and the mean age ranged from 59.62 to 69.01 years. The duration of disease ranged from 3.8 to 9.9 years, while among healthy controls, the sample ranged from 15 to 198. The sex ratio (female/male) of controls ranged from 11.11% to 150% and their mean age ranged from 58 to 68.3 years.

Likewise, measurement methods varied among different studies, including ELISA, Luminex, and others (electrochemiluminescence, restriction enzyme analysis, enzyme colorimetry). Twelve out of 13 studies described the total salivary alpha‐synuclein among PD and controls, while one study had unclear data.^11^ Only four studies described salivary oligomeric α‐syn level and oligomeric to total ratio. The details of different methods used and outcomes quantified are tabulated in Table 1.

### 3.3 NOS scale

The quality assessment done for observational studies by the NOS scale showed the scores ranged from 6 to 8. (Appendix S2). All the studies were included in the systematic review and meta‐analysis.

### 3.4 Total salivary alpha‐synuclein

**FIGURE 2:**
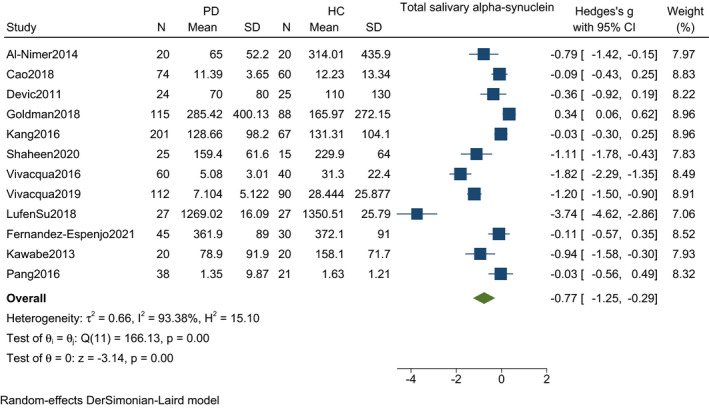
Forest plot with 95% CI for meta‐analysis on the standardized mean difference of total salivary alpha‐synuclein among PD and controls. The square shows the mean difference for each study. The diamond at the bottom of the graph shows the average effect size of included studies

**FIGURE 3:**
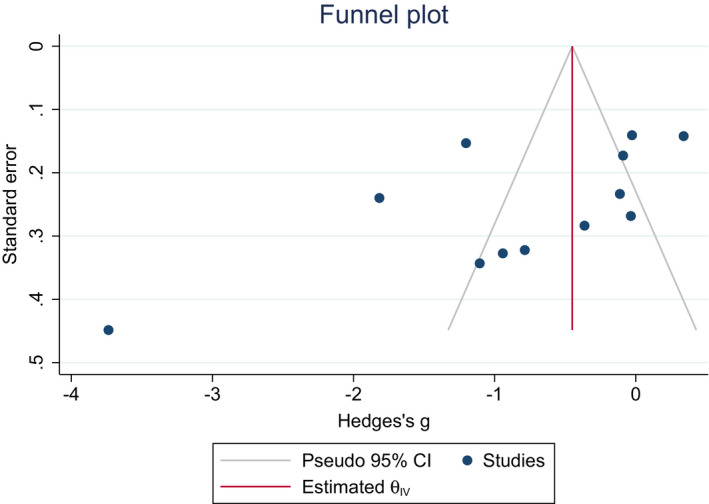
Funnel plot for detection of publication bias in meta‐analysis of standardized mean difference of total salivary alpha‐synuclein among PD and controls. Black dots represent imputed studies

Twelve studies including 761 PD patients and 503 controls found a significant reduction in a mean difference of total salivary alpha‐synuclein among PD patients as compared to healthy controls [SMD (Hedges' g = −0.77, 95% CI: −1.25 to −0.29, *p*‐value = <0.001). A random‐effect model was used that showed significant heterogeneity (*I* ^2^ = 93.38%, *p*‐value = <0.001) (Figure 2 and 3).

### 3.5 Oligomeric salivary alpha‐synuclein

**FIGURE 4:**
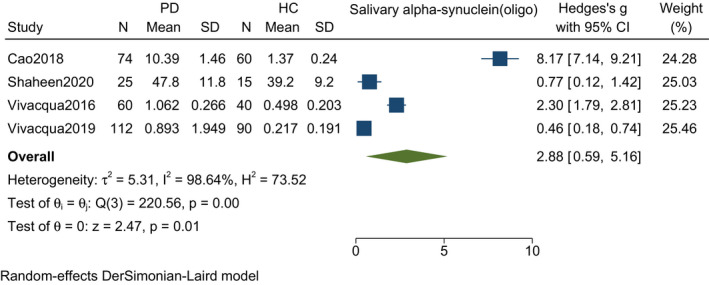
Forest plot with 95% CI for meta‐analysis on the standardized mean difference of oligomeric salivary alpha‐synuclein among PD and controls. The square shows the mean difference for each study. The diamond at the bottom of the graph shows the average effect size of included studies

Four studies including 271 PD patients and 205 controls found a significant increase in the mean difference of oligomeric salivary alpha‐synuclein among PD patients as compared to healthy controls [SMD (Hedges' g) = 2.88, 95% CI: 0.59–5.16, *p*‐value = <0.001). A random‐effect model was used that showed significant heterogeneity (*I* ^2^ = 98.64%, *p*‐value = 0.01) (Figure 4).

### 3.6 Oligomeric/total salivary alpha‐synuclein

**FIGURE 5:**
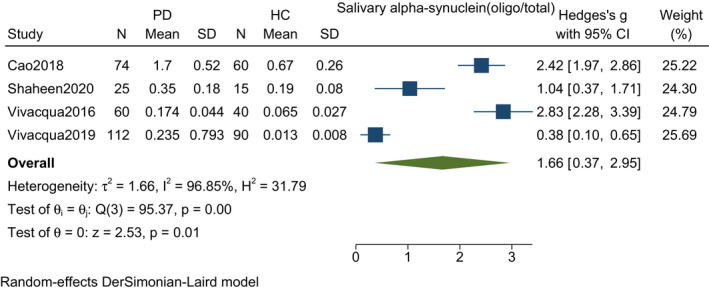
Forest plot with 95% CI for meta‐analysis on the standardized mean difference of oligo to total salivary alpha‐synuclein among PD and controls. The square shows the mean difference for each study. The diamond at the bottom of the graph shows the average effect size of included studies

Four studies including 271 PD patients and 205 controls found a significant increase in the mean difference of oligomeric to total salivary alpha‐synuclein ratio among PD patients compared to healthy controls [SMD (Hedges' g = 1.66, 95% CI: 0.37–2.95, *p*‐value = 0.01). A random‐effect model was used that showed significant heterogeneity (*I* ^2^ = 96.85%, *p*‐value = <0.001) (Figure 5).

### 3.7 Subgroup analysis, meta‐regression

To explore the cause of heterogeneity, subgroup analysis and meta‐regression were done comparing mean total salivary alpha‐synuclein among PD and healthy controls on various subheadings based on country of study, measurement methods, sex ratio, and disease duration. The subgroup analysis is described in Table 2. The subgroup difference was significant for disease duration(*p *= 0.028) while not significant for the country of study (*p *= 0.089), methods of measurement (*p *= 0.055) and sex ratio (*p *= 0.555).

**TABLE 2:** Subgroup analysis for included studies

Subgroups	No. of studies	ES	LCI	UCI	I2	*P*‐value	Subgroup difference
Method of measurement							
ELISA	7	−1.159	−1.977	−0.341	95.70%	0.005	0.055
Luminex	2	−0.105	−0.383	0.174	11.46%	0.461	
Others	3	−0.303	−0.792	0.186	66.15%	0.224	
Country of study							
China	4	−0.718	−2.218	0.692	97.45%	<0.001	0.089
Italy	2	0.073	−0.154	0.299	0%	0.997	
USA	2	−0.369	−0.782	0.044	48.91%	0.162	
Others	4	0.361	−0.13	0.852	65.14%	0.035	
Sex ratio							
<50%	2	−0.548	−0.965	−0.13	0.00%	0.01	0.555
>50%	9	−0.804	−1.398	−0.21	95.08%	0.008	
Disease duration							
≤7 years	4	−0.941	−1.807	−0.074	91.54%	0.033	0.028
>7 years	3	0.005	−0.426	0.436	68.12%	0.982	

Meta‐regression was done among 12 studies under the above subgroup headings. We found that country of study (*p *= 0.323), methods of measurement (*p *= 0.076), sex ratio (*p *= 0.459), and disease duration (*p *= 0.899) did not significantly affect effect sizes of mean differences among PD and healthy controls (Appendix S3).

### 3.8 Sensitivity analysis and publication bias

A sensitivity test performed by sequentially omitting one study at a time and recalculating the summary effect size showed that the recalculated effect size was adamant, indicating the stability of the analysis. Similarly, recalculated heterogeneity was also similar across studies.

The funnel plot and Begg's test were conducted to assess the potential publication bias. No publication bias was observed (*p *= >0.05) (Figure 2).

The number of studies for other outcomes like oligomeric salivary alpha‐synuclein and oligo to total ratio of salivary alpha‐synuclein was not sufficient for performing subgroup analysis, meta‐regression analysis, and publication bias (less than 5).

## 4 DISCUSSION

This systematic review and meta‐analysis consisted of a total of 13 studies assessing the association of salivary alpha‐synuclein levels and Parkinson's disease. A previous systematic review by Bougea et al. comprising eight studies found that the diagnostic performance of the salivary synuclein was not robust enough to support the diagnosis of Parkinson's disease.^21^ The pooled result showed the significant reduction of total salivary alpha‐synuclein levels while significant increment in oligomeric and oligomeric to total salivary alpha‐synuclein levels among PD patients compared to controls.

It is well known that alpha‐synuclein makes a significant part of the protein content of LB associated with Parkinson's disease, and the aggregates of higher molecular weight causing cellular toxicity drive the disease process.^22^, ^23^ The neuropathological studies have shown that in the early stages of the disease, α‐Syn intracellular aggregates mainly via exosomes in several brainstem nuclei, including the superior and inferior salivary nuclei and the parasympathetic salivary ganglia.^24^ There is also the prion‐like propagation from neuronal cell bodies of salivary neurons to the synaptic terminals around the epithelial cells of salivary glands.^25^ The Braak hypothesis has also suggested that α‐Syn spreads from the nose to the GI tract and then finally enters the brain via the vagus nerve or olfactory tract.^26^ Several studies have found a high concentration of α‐Syn in the submandibular gland and a 100% positivity rate among PD compared to controls.^5^, ^22^ Furthermore, hyposialorrhoea as an early presentation of PD makes the strong possibility for the contribution of the autonomic fibers in the development of α‐Syn pathology in the salivary glands.^27^ New evidence has shown that the pathogenesis in PD starts many years before the onset of motor symptoms unlocking the possibility of early clinical diagnosis.^28^ Hence, measuring salivary alpha‐synuclein parameters as biomarkers for PD seems completely justifiable.

In our meta‐analysis, total salivary alpha‐synuclein levels were significantly lower in PD than controls. However, this finding was inconsistent with many studies included in the analysis.^6^, ^11^, ^12^, ^13^, ^14^ The possible explanation is differences in the method of analysis, sample size, and disease duration at the time of enrollment.

The oligomeric and oligo to total salivary alpha‐synuclein levels showed significant increments in PD vs controls in accord with the individual studies while total α‐syn levels were lower in PD patients with respect to controls.^6^, ^8^, ^9^, ^10^ While the precise mechanism is unknown, the efflux of total α‐syn levels may be regulated differently than oligomeric α‐syn. In a model of injury by alpha‐synuclein, the oligomer is considered more toxic than the monomer. It was suggested that α‐syn oligomers might be a more accurate indicator of the disease than total α‐syn levels.^23^ The oligomer levels are elevated in the cerebral cortex and brainstem of PD patients compared to the controls.^29^ Furthermore, in CSF, oligomers measured the sensitivity and specificity to be 75.0% and 87.5%, respectively, and when the ratio of oligomers/total alpha‐synuclein was measured, it increased to 89.3% and 90.6%.^9^

Kang et al. showed a decrease in salivary alpha‐synuclein with the age of PD patients. The cause behind this may be due to undetectable epitome masked in aggregates.^13^ Our study does not reflect such findings. Stewart et al. found alpha‐synuclein higher in women than men.^11^ Our subgroup analysis showed no significant results in terms of sex ratio, yet most of the studies had female predominant PD patients. Shi et al. showed that alpha‐synuclein in blood exosomes are correlated significantly with disease severity in patients with PD, while Shaheen et al. showed similar findings in saliva.^10^, ^30^ Kang et al. failed to show an association of salivary alpha‐synuclein levels with disease stages and motor symptoms.^13^ The subgroup analysis showed significant findings for lower disease duration, but the findings cannot be generalized because of the inconsistent cut‐off value for disease duration. Ethnicity also plays a vital role in the genetic susceptibility of PD, like polymorphism of the SNCA gene.^13^ But our study did not show significant differences among the patients residing in different countries. The coexistence and limitation of different assays (ELISA vs Luminex vs others) showed contrasting results. Two studies showed no significant results by Luminex,^11^, ^14^ while others showed significant results by ELISA.^7^, ^8^ Our study showed no significant subgroup differences for the assays used.

CSF, blood, nerve plexus, gut mucosa, and skin biopsy have been tested for alpha‐synuclein levels in search of potential biomarkers. Although CSF a‐syn levels produce the most consistent results, it is invasive. It may not be feasible to monitor long‐term progression and challenging to implement in a center with less experience. Skin biopsy, gut mucosa, and nerve plexus require an invasive procedure and have produced inconsistent results. Blood is easily accessible, but its level in blood is influenced by the degree of hemolysis and hence may not be suitable for diagnostic purposes.^31^ Body fluid biomarkers like CSF, blood, and saliva are superior to clinical features for diagnosing PD as biases less influence them. They may reduce the misdiagnosis of PD by approximately 20%.^32^ Among all salivary samples have various advantages. They are relatively safe, easily accessible, and less invasive with low blood contamination levels to adequately collect DNA, microRNA, protein, and metabolite analyses.^33^ Vivacqua et al. has also shown a relative cut‐off value with high specificity and sensitivity to differentiate PD patients and controls.^9^ Thus, salivary alpha‐synuclein is our best choice among all the fluid biomarkers for early diagnosis and progression of the disease.

Early diagnosis can also unlock the possibility of advancement of new therapeutic interventions for the treatment of PD at an early stage.^28^ A neuroprotective therapy administered early is helpful in PD patients. However, a clinically helpful drug is not available to date when PD is identified unequivocally at a very early stage, only then identifying and validating a neuroprotective drug through randomized control trial.^34^ Thus, salivary biomarkers can play a pivotal role in these. However, the future is challenging; fluid biomarkers remain less sure than neuroimaging and tissue biopsy.^21^ Beyond all the above, there is growing interest in digital biomarkers like portable smartphones to identify the earliest prodromal PD, providing reliable disease progression in trials.^35^

### 4.1 Strengths and limitations

This meta‐analysis includes data from 12 studies to show the pooled mean differences of salivary α‐syn levels among PD and controls. The findings of the present study have significant clinical and research implications. There were no language barriers, so studies with different ethnicity were included. We did subgroup analyses and meta‐regression to find the cause of heterogeneity.

There are several limitations of our study. The studies included in our systematic review and meta‐analysis included PD patients at different levels of severity. There are great varieties among studies selected for this article causing high‐level heterogeneity, which might be due to the coexistence of various variables like α‐syn species (total vs oligomeric), assays (ELISA vs Luminex), disease stage, genetic status, and patient groups. The diagnostic accuracy of salivary alpha‐synuclein is not described in our study. Similarly, there is no clear gold standard protocol for saliva collecting procedure, and microbiome composition may be influenced by the type of saliva collected (stimulated or unstimulated), questioning the conclusion based on biomarkers.^36^, ^37^ Finally, our study does not answer what population group should be targeted for such an intervention. Studies have shown that patients develop nonmotor symptoms years before developing motor symptoms. Our study cannot establish the utility of salivary α‐syn levels in a healthy population.

Further, there is significant variation among studies regarding disease duration and α‐syn levels. Hence, its utility in monitoring disease progression cannot be established now. It is imperative that future studies employ uniform testing methods, and include patients with stringent inclusion criteria and at the same stage or duration of PD diagnosis.

## 5 CONCLUSION

Our systematic review and meta‐analysis found that salivary alpha‐synuclein parameters (total, oligomeric, oligo/total) were significantly different between PD patients and controls, suggesting usefulness as a simple, easy to use, cost‐effective, and reliable diagnostic biomarker for PD and its progression. Further studies, preferably with a larger sample size and multi‐ethnic, uniform diagnostic assay, and disease severity are warranted. These factors may help us evaluate the utility of measuring salivary alpha‐synuclein in diagnosing PD and monitoring its progression along with differentiation from other neurodegenerative disorders.

## CONFLICT OF INTEREST

The authors declare no conflicts of interest.

## AUTHOR CONTRIBUTIONS

SK and RO designed the study. SK and AB carried out the literature search, review, and selection. SK carried out the statistical analysis. SK, AB, and SPJ drafted the manuscript. RR, JKY, and RO were critically revising the manuscript for important intellectual content. All authors read and approved the final manuscript.

## Supporting information

Appendix S1‐S3
