# Supplementary material for: Salivary alpha‐synuclein as a potential fluid biomarker in Parkinson’s disease: A systematic review and meta‐analysis
Source: Aging Med (Milton). 2022 Jan 24;5(1):53–62. doi: 10.1002/agm2.12192 (PMC8917264; doi:10.1002/agm2.12192)
Supplement: Supplementary file 1 — Appendix S1‐S3 [file AGM2-5-53-s001.docx]

Supplementary Content

**Appendix 1:** Search strategy used in the current systematic review and meta-analysis.

**Appendix 2:** Quality assessment of the included observational articles.

**Appendix 3**: Meta-Regression for the study outcome

This supplementary material has been provided by the authors to give readers additional information.

**Appendix 1:** Search strategy used in the current systematic review and meta- analysis.

**For PubMed**

#1: ("Parkinson Disease"[Mesh]) OR ("Parkinsonian Disorders"[Mesh])

#2: "alpha-Synuclein"[Mesh] AND ("Saliva" OR "Salivary gland") OR "Salivary alpha-synuclein"

#3: #1 AND #2

Filters: human subjects, Time frame from 2010 to 2021

# Final search strategy after filters:

# ("Parkinson Disease"[MeSH Terms] OR "Parkinsonian Disorders"[MeSH Terms]) AND (("alpha-Synuclein"[MeSH Terms] AND ("Saliva"[All Fields] OR "Salivary gland"[All Fields])) OR ("salivary"[All Fields] AND ("alpha-Synuclein"[MeSH Terms] OR "alpha-Synuclein"[All Fields] OR ("alpha"[All Fields] AND "synuclein"[All Fields]) OR "alpha-Synuclein"[All Fields] AND (humans[Filter]) AND (2010:2021[pdat]))

# Total studies: 45

# Embase search strategy

# #1: ’alpha synuclein’/mj

# #2: ’salivary alpha synuclein’

# #3: ‘parkinson disease’/exp

# #4: ‘parkinsonism’/exp

# #5: ‘parkinson’

# #6: ‘saliva’

# #7: ‘salivary gland’

# #8: #6 OR #7

# #9: #1 AND #8

# #10: #2 OR #9

# #11: #3 OR #4 OR #5

# #12: #10 AND #11

# #13: #10 AND #11 AND [humans]/lim

**Appendix 2:** Quality assessment of the included observational articles.

| Study Name | Selection | Comparability | Outcome | Total Score |
| --- | --- | --- | --- | --- |
| Al-Nimer2014 et al. | 3 | 1 | 3 | 7 |
| Cao2018 et al. | 3 | 2 | 3 | 8 |
| Devic2011 et al. | 3 | 1 | 3 | 7 |
| Goldman2018et al. | 3 | 2 | 3 | 8 |
| Kang2016et al. | 3 | 2 | 3 | 8 |
| Shaheen2020 et al. | 3 | 1 | 3 | 7 |
| Stewart2014 et al. | 3 | 1 | 3 | 7 |
| Vivacqua2016 et al. | 3 | 1 | 3 | 7 |
| Vivacqua2019 et al. | 3 | 2 | 3 | 8 |
| Lufen Su2018 et al. | 3 | 1 | 3 | 7 |
| Fernandez-Espejo2021 et al. | 3 | 2 | 3 | 8 |
| Kawabe2013 et al. | 2 | 1 | 3 | 6 |
| Pang2016 et al. | 3 | 1 | 3 | 7 |

# Note: Mean scores greater or equal to 5 are included in analysis.

**Appendix 3**: **Meta-Regression for the study outcome**

| Variables | Coefficient | Standard Error | z | p>\|z\| | 95% (C.I) | |
| --- | --- | --- | --- | --- | --- | --- |
| Method of measurement | 0.6764379 | 0.3810607 | 1.78 | 0.076 | -.0704284 | 1.423302 |
| Country of study | 0.2547839 | 0.2575661 | 0.99 | 0.323 | -.2500362 | .7596041 |
| Sex ratio | -0.501503 | 0.6776208 | -0.74 | 0.459 | -1.829615 | . 8266094 |
| Disease duration | -0.496641 | 0.3900425 | -0.13 | 0.899 | -.8141333 | .7148052 |
| Constant | -1.485014 | 1.503675 | -0.99 | 0.323 | -4.432164 | 1.462135 |
